# Joint synthesis of conditionally related multiple outcomes makes better use of data than separate meta‐analyses

**DOI:** 10.1002/jrsm.1380

**Published:** 2019-11-10

**Authors:** Sumayya Anwer, A.E. Ades, Sofia Dias

**Affiliations:** ^1^ Centre for Reviews and Dissemination University of York YO10 5DD UK; ^2^ Bristol Medical School, University of Bristol Canynge Hall, 39 Whatley Road, BS8 2PS UK

**Keywords:** Bayesian, Early onset group B streptococcus, Intrapartum antibiotic prophylaxis, Meta‐analysis, Multi‐outcome synthesis, Multi‐state models

## Abstract

**Background:**

When there are structural relationships between outcomes reported in different trials, separate analyses of each outcome do not provide a single coherent analysis, which is required for decision‐making. For example, trials of intrapartum anti‐bacterial prophylaxis (IAP) to prevent early onset group B streptococcal (EOGBS) disease can report three treatment effects: the effect on bacterial colonisation of the newborn, the effect on EOGBS, and the effect on EOGBS conditional on newborn colonisation. These outcomes are conditionally related, or nested, in a multi‐state model.

This paper shows how to exploit these structural relationships, providing a single coherent synthesis of all the available data, while checking to ensure that different sources of evidence are consistent.

**Results:**

Overall, the use of IAP reduces the risk of EOGBS (RR: 0.03; 95% Credible Interval (CrI): 0.002–0.13). Most of the treatment effect is due to the prevention of colonisation in newborns of colonised mothers (RR: 0.08, 95% CrI: 0.04–0.14). Node‐splitting demonstrated that the treatment effect calculated using only direct evidence was consistent with that predicted from the remaining evidence (*p* = 0.15). The findings accorded with previously published separate meta‐analyses of the different outcomes, once these are re‐analysed correctly accounting for zero cells.

**Conclusion:**

Multiple outcomes should be synthesised together where possible, taking account of their structural relationships. This generates an internally coherent analysis, suitable for decision making, in which estimates of each of the treatment effects are based on all available evidence (direct and indirect). Separate meta‐analyses of each outcome have none of these properties.

**HIGHLIGHTS**

**What is already known?**

Most previous estimates for the effectiveness of Intrapartum antibiotic prophylaxis for early onset group B streptococcal (EOGBS) disease were biased due to inappropriate methods being applied to meta‐analyse studies with strong treatment effects and a large number of zero cells.

**What is new?**

Synthesis using multi‐state models delivers a single coherent analysis of multiple evidence sources using conditional relationships. The relative treatment effects estimated are more precise than when separate analyses were conducted.

**Potential impact for Review Synthesis Methods readers outside the authors' field**

Wherever possible, multiple outcomes should be synthesised together in a single coherent analysis, capturing the clinical and structural relationships between them. A single set of coherent estimates improves the robustness and allows a better understanding of the effectiveness of the intervention, facilitating decision‐making.

## 1 INTRODUCTION

Health technology assessments are carried out to evaluate the efficacy of medical interventions, and inform a decision of whether to use them for a particular group of patients. Usually these assessments rely on a systematic review of the literature, followed by a meta‐analysis. It is considered good practice to define the main review outcome *a priori* with other outcomes of interest classified as secondary and each outcome analysed separately.^1^, ^2^, ^3^ However, often outcomes are known to be related. A joint synthesis of all related outcomes, where their relationship (including any correlations) is taken into account, is preferable as it will use all the relevant evidence in a single coherent analysis.

One approach to multiple outcome synthesis has been multivariate normal random effects (MVNRE) models taking account of correlations between outcomes, both within and between studies.^4^, ^5^ Alternatively, multi‐parameter evidence synthesis (MPES) uses different data sources to inform parameters which are related in a mathematical model,^6^, ^7^, ^8^ thus capturing structural and logical relationships between outcomes, and generating outputs that have a natural clinical interpretation.

In this paper, we present a particular type of multiple outcome data where outcomes are nested, that is, some events can only occur in individuals who already had a previous (related) event. In other words, there is a logical chain of events which occur in a known sequence, with each outcome conditional on the occurrence of the previous outcome. We will present an illustrative example where evidence on such conditional outcomes is available, forming a multi‐state model. We will show how to incorporate all the outcomes into a single meta‐analysis and how to check for potential conflict between evidence sources. Finally, we highlight the benefits of our approach compared to previous meta‐analysis models.

## 2 ILLUSTRATIVE EXAMPLE: EARLY ONSET GROUP B STREPTOCOCCAL DISEASE

**Figure 1:**
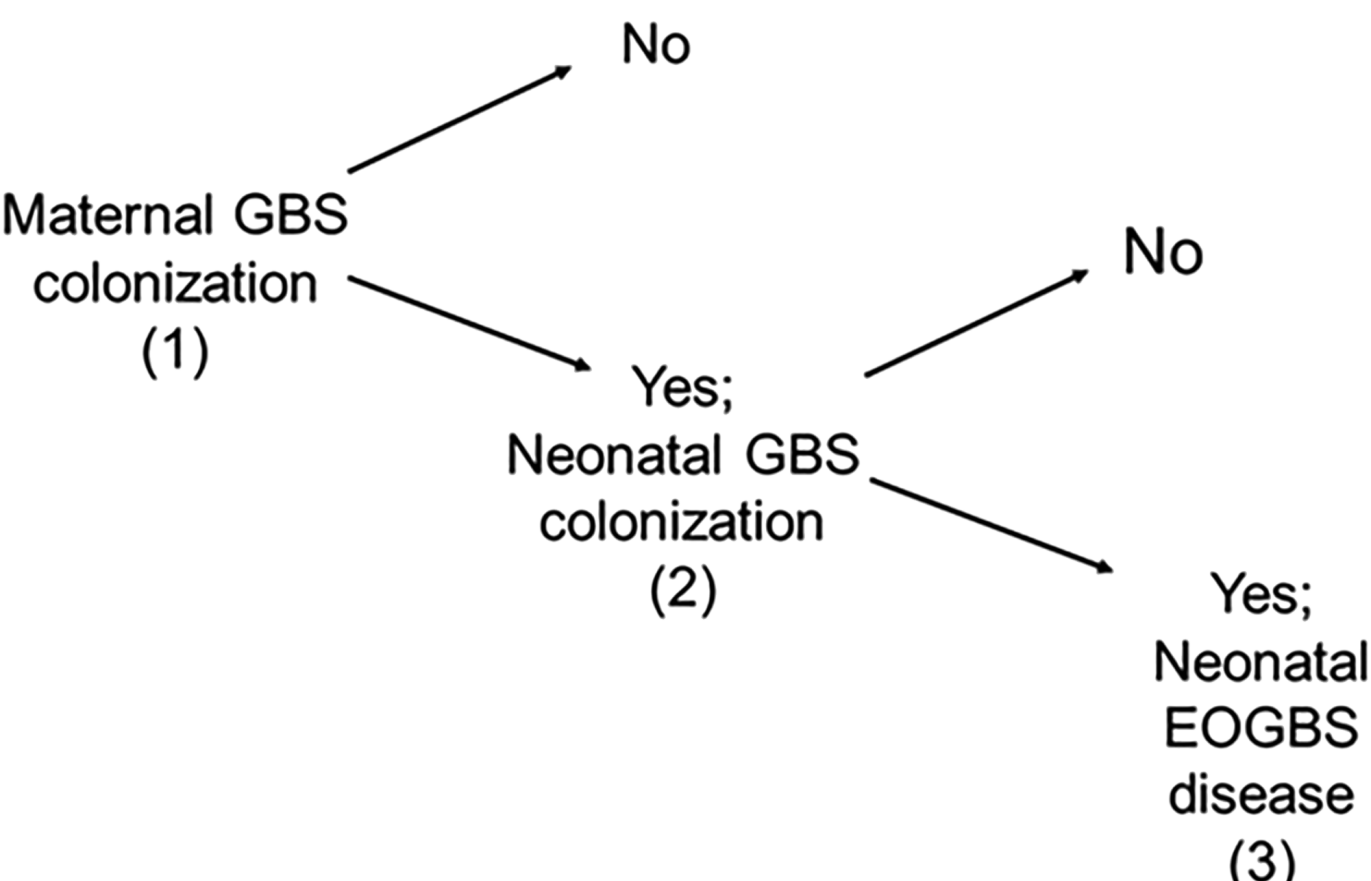
Multi‐state model structure for the EOGBS example

Our study looks at trials of intrapartum antibiotic prophylaxis (IAP) to prevent early onset group B streptococcal (EOGBS) disease in newborns. Newborns can probably only develop EOGBS if they have been colonised by the bacteria, which in turn is only possible if the mother was a group B streptococcus (GBS) carrier during labour^9^, ^10^ (Figure 1).

Maternal colonisation with GBS is relatively common, averaging 18% but varying from 11%‐ 35% in different countries.^11^ EOGBS occurs in 0.43 per 1000 births world‐wide,^12^ with wide variation between regions. EOGBS is associated with high rates of meningitis and neonatal encephalopathy, with a 12.1% case fatality.^12^ At least 60 countries have adopted preventative strategies, ranging from IAP for women identified at high risk to universal screening for maternal carriage and IAP in those screened positive.^13^ The efficacy of IAP is widely recognised based both on randomised control trials and on a range of observational data.^14^ However, the best preventative strategy remains controversial.^15^, ^16^, ^17^

Several trials have been conducted on women colonised with GBS; some have reported neonatal colonisation as the outcome, some neonatal EOGBS disease, and others both. We number these outcomes (or states) maternal colonisation *(1)*, newborn colonisation *(2)*, and EOGBS *(3).*

A change in state is referred to as a transition.^18^, ^19^, ^20^ The transition between states are labelled as 1 → 2, 2 → 3 and 1 → 3. Table 1 reports all the aggregate data available from eight studies^21^, ^22^, ^23^, ^24^, ^25^, ^26^, ^27^, ^28^ that compare the effectiveness of IAP administered to mothers intravenously to a placebo or control. Multiple births were rare in the data, and therefore the lack of independence between twins in the data was ignored.^29^ Studies 1–3, 6 and 8 report both infant colonisation and EOGBS outcomes, and thus provide evidence on all three transitions. Studies 4 and 5 report only infant colonisation and thus provide evidence on the 1 → 2 transition only. Study 7 only provides evidence on the proportion of patients making both a 1 → 2 and 2 → 3 transition. Standard meta‐analytic methods have been applied to both the 1 → 2^30^ and 1 → 3^9^, ^30^, ^31^, ^32^ effects. The difficulty with having separate, unrelated meta‐analyses is that these are not independent as some of the trials are involved in both and because the 1 → 2 effect is part of the 1 → 3 effect. Also, it is unclear how one can draw an overall conclusion from the two sets of estimates produced.

**Table 1:** Study details for intrapartum antibiotic prophylaxis for the prevention of EOGBS in newborns. The number of individuals experiencing an event *x* (where 1 = maternal colonisation, 2 = neonatal colonisation and 3 = EOGBS) in a study *i* for the placebo (*k = 1)* and IAP *(k = 2)* arms are denoted by *r*_*x*,*ik*_

	Maternal colonisation (1)		Neonatal colonisation (2)		EOGBS (3)	
	Placebo	IAP	Placebo	IAP	Placebo	IAP
	*r*_1, *i*1_^*^	*r*_1, *i*2_^*^	*r*_2, *i*1_	*r*_2, *i*2_	*r*_3, *i*1_	*r*_3, *i*2_
1. Boyer (1982) 21	82	69	46	2	4	0
2. Boyer (1983) 22	37	43	13	1	1	0
3. Matorras (1991) 23	56	54	24	2	3	0
4. Easmon (1983) 24	49	38	17	0		
5. Yow (1979) 25	24	34	14	0		
6. Morales (1986) 26	128	135	59	0	2	0
7. Tuppurainen (1989) 27	111	88			4	1
8. Boyer (1986) 28	79	85	40	8	5	0

* These are the total number of individuals that were randomised.

In addition, relevant evidence from two studies (4^20^ and 5^21^ in Table 1) would be excluded from a meta‐analysis where the outcome of interest was EOGBS given maternal colonisation. The large number of zero counts in the IAP treatment arm (because the treatment is very effective), adds further complexity to the analysis.

## 3 METHODS

### 3.1 Data and likelihoods

Let *r*_*x* → *y*,*ik*_ denote the number of individuals with event of type *y* given previous state *x* in arm *k* of trial *i* where *x* = 1, 2 represents maternal colonisation or neonatal colonisation and *y* = 2, 3 represents neonatal colonisation and EOGBS, respectively (Figure 1, Table 1). The likelihoods conditional on a previous outcome are binomial:

(1) $$r_{x\to y,\mathrm{ik}}\sim \text{Binomial}\left(\pi_{x\to y,\mathrm{ik}},n_{x,\mathrm{ik}}\right)$$

where *π*_*x* → *y*,*ik*_ represent the conditional probabilities of achieving outcome *y* = 2, 3 for individuals in state *x* = 1, 2, that is the probability of transitioning from 1 → 2, 2 → 3 or 1 → 3; and *n*_*x*,*ik*_ represent the number of individuals in state *x*, that is *n*_1,*ik*_, the number of women colonised with GBS and *n*_2,*ik*_ the number of colonised newborns in arm *k* of trial *i.*

The likelihoods in Equation (1) take into account the information available in each study.

For example, studies 1–5^21^, ^22^, ^23^, ^24^, ^25^ and 8^28^ provide data on neonatal colonisation *(2)* given maternal colonisation *(1).* Therefore, we can estimate the probability of transitioning from 1 → 2 in each of these studies by specifying a likelihood for *r*_1 → 2,*ik*_ using the total number of colonised women as the denominator.

Study 7^27^ provides information only on EOGBS *(3)* given maternal colonisation *(1)* thus we can estimate the probability of transitioning from 1 → 3 in this study by specifying a likelihood for *r*_1 → 3,*ik*_ using the total number of colonised women as the denominator.

Additionally, studies 1–3^21^, ^22^, ^23^ and 8^28^ also provide data on EOGBS *(3).* Rather than use this data to estimate the 1 → 3 probabilities, which would involve “double counting” as it is not independent of the 1 → 2 data, we can instead estimate the probability of transitioning from 2 → 3 by specifying a likelihood for *r*_2 → 3,*ik*_ conditioning on the total number of colonised newborns *n*_2,*ik*_ as the denominator. These studies contribute indirect evidence on the probability of transitioning from 1 → 3 (see Section 3.2).

Study 6^26^ was used to inform the overall treatment effect (1 → 3) instead of using the results to inform transitions 1 → 2 and 2 → 3 separately as it effectively provides no information on the latter transition, since no newborns in the IAP arm were colonised (leading to a denominator of 0 when describing the 2 → 3 transition). Therefore, six studies provide evidence to the 1 → 2 transition, four to the 2 → 3 transition, and 2 to the 1 → 3 transition for the multiple‐outcome meta‐analysis model for EOGBS.

To combine all data in a single, coherent analysis, we need to express the relationship between the relative treatment effects on the 1 → 2, 2 → 3, and 1 → 3 transitions.

### 3.2 Relationships between states

A relationship can be established between the Relative Risks (RRs) estimated from the three sources of evidence. The RR for the 1 → 2 transition is defined as:

(2) $$\mathrm{RR}\left(1\to 2\right)=\frac{\mathrm{Pr}\left(1\to 2|T\right)}{\mathrm{Pr}\left(1\to 2|C\right)}$$

where Pr(1 → 2| *T*) and Pr(1 → 2| *C*) are the probabilities of neonatal colonisation conditional on maternal colonisation under IAP and control, respectively.

Similarly, the RR for the 2 → 3 transition is defined as:

(3) $$\mathrm{RR}\left(2\to 3\right)=\frac{\mathrm{Pr}\left(2\to 3|T\right)}{\mathrm{Pr}\left(2\to 3|C\right)}$$

Assuming the transitions 1 → 2 and 2 → 3 are conditionally independent, it follows that:

(4) $$\begin{array}{lll} \mathrm{RR}\left(1\to 2\right)\cdot \mathrm{RR}\left(2\to 3\right) & = & \frac{\mathrm{Pr}\left(1\to 2|T\right)}{\mathrm{Pr}\left(1\to 2|C\right)}\cdot \frac{\mathrm{Pr}\left(2\to 3|T\right)}{\mathrm{Pr}\left(2\to 3|C\right)}\\ & = & \frac{\mathrm{Pr}\left(1\to 3|T\right)}{\mathrm{Pr}\left(1\to 3|C\right)}\\ & = & \mathrm{RR}\left(1\to 3\right) \end{array}$$

We define the RR on the 1 → 2 and 2 → 3 transitions as the basic parameters^33^, ^34^ to be estimated and will impose the constraint *RR*(1 → 3) = *RR*(1 → 2) ⋅ *RR*(2 → 3) (Equation (4)). Note that no such relationship exists if the treatment effect is defined in terms of the Risk Difference or Odds Ratio.

### 3.3 Meta‐analysis models

#### 3.3.1 Multi‐state model

A Bayesian multi‐state model (referred to as the base‐case model) estimates the log relative risks (LRRs) whilst ensuring that estimated probabilities remain between zero and one,^35^ and incorporating Equation (4).

Using the likelihood defined in Equation (1), the LRRs for the control and treatment arms for any transition *x* → *y,* are modelled as:

(5) $$\begin{array}{l} \log\left(\pi_{x\to y,i1}\right)=\mu_{x\to y,i}\\ \log\left(\pi_{x\to y,i2}\right)=\mu_{x\to y,i}+\min\;\left(\delta_{x\to y,i},-\mu_{x\to y,i}\right) \end{array}$$

For a trial *i*, *μ*_*x* → *y*,*i*_ is the log of the probability of the transition in the control arm of trial *i*, which is given a non‐informative prior distribution, Uniform (0,1) , and considered a nuisance parameter.^35^ At the same time the LRR, *δ*_*x* → *y*,*i*_, is constrained to ensure probabilities remain between zero and 1.^35^, ^36^

Then, in an FE model *δ*_*x* → *y*,*i*_ = *d*_*x* → *y*_, while for an RE model, we write:

(6) $$\delta_{x\to y,i}\;\sim \;\text{Normal}\;\left(d_{x\to y},\;,\sigma_{x\to y}^{2}\right)$$

where *d*_*x* → *y*_ is the mean treatment effect and $\sigma_{x\to y}^{2}$ the between‐study heterogeneity variance. The RE model was used to model the LRR of neonatal colonisation on maternal colonisation (transition 1 → 2) as:

(7) $$\begin{array}{l} \delta_{1\to 2,i}\;\sim \;\text{Normal}\left(d_{1\to 2},\;\sigma_{1\to 2}^{2}\right)\\ d_{1\to 2}\;\sim \;\text{Normal}\left(0,\;1000\right)\\ \sigma_{1\to 2}\;\sim \;\text{Half}-\text{Normal}\left(0,\;0.32^{2}\right) \end{array}$$

The half‐normal prior distribution for the between‐trial standard deviation generates only positive values, and its variance is chosen so that the 95% Credible Interval (CrI) for the effects of trials lies within a factor of 2 from the median.^36^

The LRR of EOGBS conditional on neonatal colonisation (transition 2 → 3) is modelled using a FE model due to data sparseness:

(8) $$\delta_{2\to 3,i}=d_{2\to 3}\;\sim \;\text{Normal}\left(0,\;10\right)$$

The relationship in Equation (4), on the log‐scale, is used to describe the overall treatment effect (transition 1 → 3), as the sum of a random effect for the 1 → 2 transition and a fixed effect for 2 → 3:

(9) $$\begin{array}{l} \delta_{1\to 3,i}\;\sim \;\text{Normal}\left(d_{1\to 3},\sigma_{1\to 3}^{2}\right)\\ d_{1\to 3}=\;d_{1\to 2}+\;d_{2\to 3} \end{array}\text{.}$$

This captures the assumption that the relative treatment effect for 2 → 3, *d*_2 → 3_, has a fixed effect, forcing the variance of 1 → 3 to be the same as the variance of 1 → 2.

#### 3.3.2 Sensitivity analyses

Modelling assumptions regarding the way trials 6 and 7 were incorporated in the base‐case model were investigated in sensitivity analyses. In a second multi‐state model, Sensitivity Analysis (SA) 1, the overall treatment effect is modelled using a FE model where *δ*_1 → 3,*i*_ = *d*_1 → 3_ with *d*_1 → 3_ defined in Equation (9). A third model, SA 2, assumed that all the effect of IAP in EOGBS is achieved through preventing neonatal colonisation: thus we set *d*_2 → 3_ = 0, resulting in *d*_1 → 3_ = *d*_1 → 2_.

We also examined more informative and less informative prior distributions for the between‐trial standard deviation *σ*_1 → 2_. These were Half‐Normal (0, 0.19^2^) and Half‐Normal (0, 0.50^2^), which imply that 95% of the trial effects are within a factor of 1.5 and 3.0 from the median, respectively.

A t‐distribution prior with a mean of zero and two degrees of freedom was used to investigate the sensitivity of the prior for *d* (Supplementary Figures S2 and S3).

#### 3.3.3 Standard meta‐analysis models

Standard Bayesian meta‐analysis models were also applied, using the same priors as the multi‐state models; an RE model for the 1 → 2 transition and both FE and RE models for the 2 → 3 and 1 → 3 transitions using a binomial likelihood,^37^, ^38^ in each case using all the data available on each transition. These analyses are not independent but are presented for comparison.

### 3.4 Model estimation

Models were estimated by Markov Chain Monte Carlo (MCMC) methods in WinBUGS 1.4.3.^39^ The multi‐state structures are implemented by adapting the code given in Dias, Ades, Welton, Jansen, Sutton 36 for ‘chains of evidence’ structures, included in the Supplementary Files (Supplementary File S4).

Convergence was assessed as having occurred within 15,000 iterations using the Brooks‐Gelman‐Rubin (BGR) diagnostic 40 and trace plots. We discarded the first 200,000 samples (burn in), and based inference on 100, 000 samples from each of four chains.

#### 3.4.1 Model fit

The fit of the models is checked using the total residual deviance and by inspecting deviance plots.^41^ The residual deviance is the posterior mean of the deviance of the model removing the deviance for a saturated model.^42^ For models that fit the data well, the residual deviance is expected to be close to the number of unconstrained data points. Model comparison can be conducted by using DIC to compare the base‐case model, SA 1 and SA 2. The DIC measures the goodness of fit penalising for the effective number of parameters.^41^ Lower values of DIC are preferred with differences greater than 3 to 5 points, being considered important.

#### 3.4.2 Checking conflict

Node‐splitting^43^ was used to check for conflict between the “direct” evidence on the RR for the 1 → 3 transition from studies 6 and 7, and the “indirect” evidence from the rest of the data.

Therefore, we define $d_{1\to 3}^{\mathrm{Dir}}$ as:

(10) $$d_{1\to 3}^{\mathrm{Dir}}\;\sim \;\text{Normal}\;\left(0,1000\right)$$

while the “indirect” estimate is calculated as $d_{1\to 3}^{\mathrm{Ind}}=d_{1\to 2}+d_{2\to 3}$ and compared to $d_{1\to 3}^{\mathrm{Dir}}$ using a Bayesian “p‐value” 43, 44:

(11) $$p_{B}=\mathrm{Pr}\left\{d_{1\to 3}^{\mathrm{Dir}}\geq d_{1\to 3}^{\mathrm{Ind}}\right\}$$

In an MCMC framework, this p‐value is calculated as the proportion of iterations where $d_{1\to 3}^{\mathrm{Dir}}\geq d_{1\to 3}^{\mathrm{Ind}}$. If *p*_*B*_ is less than 0.5, the two‐sided p‐value is 2 × *p*_*B*_ *,* otherwise it is 2× (1‐*p*_*B*_).^43^

The fit of the node‐split model is also compared to the fit of the base‐case model using the residual deviance and DIC.^41^ The changes in between‐study heterogeneity can also be used to compare the heterogeneity of the base‐case and node‐split models.^45^

## 4 RESULTS

### 4.1 Multi‐state model

**Figure 2:**
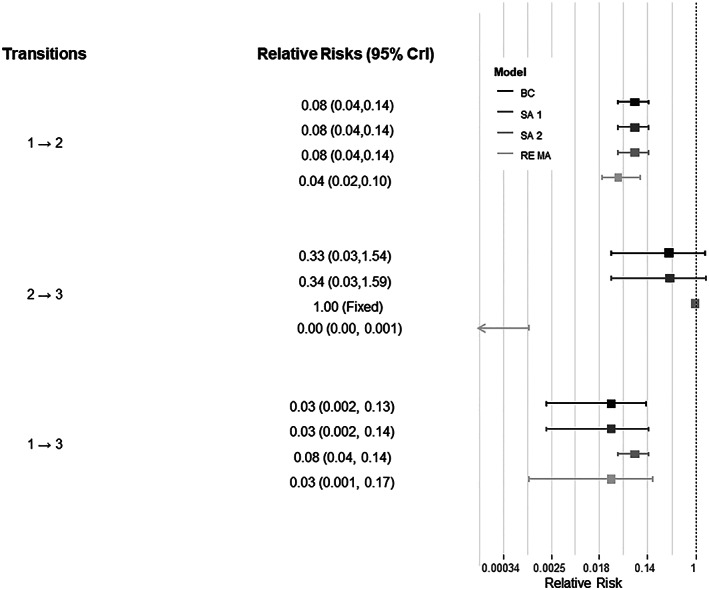
Comparative forest plots representing the relative risks and 95% Credible Intervals (CrIs) for the treatment effects of IAP on EOGBS for the (a) base‐case model (BC), (b) Sensitivity Analysis 1 (SA 1), (c) Sensitivity Analysis 2 (SA 2), and (d) standard random effects model (RE MA)

The overall treatment effect using all available relevant evidence shows that administering IAP to mothers prevents EOGBS in newborns (RR: 0.03, 95% CrI: 0.002, 0.13). The results from the base‐case model (Table 2 and Figure 2) indicate that IAP primarily prevents EOGBS disease by preventing colonised mothers from infecting their newborns, i.e. during transition 1 → 2 (RR: 0.08, 95% CrI: 0.04, 0.14). There is insufficient evidence to determine whether IAP has an additional treatment effect on preventing EOGBS in colonised newborns, i.e. transition 2 → 3 (RR: 0.33, 95% CrI: 0.03, 1.54). The residual deviance for the base‐case model is 25.5 compared to 24 data points included in the analysis, indicating a good fit.

**Table 2:** Median relative risks (RR) with 95% Credible Intervals (CrI), between‐trials standard deviance (σ) for the 1 → 2 transition, and model fit statistics for the base‐case model and sensitivity analysis. All 8 studies in Table 1 were included in each model

Model		Residual deviance†	DIC	σ_1 → 2_ (95% CrI)	RR_1 → 2_ (95% CrI)	RR_2 → 3_ (95% CrI)	RR_1 → 3_ (95% CrI)
	**Base‐case**						
Base‐case	*δ*_1 → 3,*i*_ = *δ*_1 → 2,*new*_+*d*_2 → 3_	25.5	96.3	0.278 (0.011, 0.765)	0.081 (0.039, 0.143)	0.331 (0.026, 1.538)	0.026 (0.002, 0.130)
	**Sensitivity analyses: Modelling assumptions**						
SA 1	$\begin{array}{c} \delta_{1\to 3,i}=d_{1\to 3}\\ \;=d_{1\to 2}+d_{2\to 3} \end{array}$	25.7	96.5	0.273 (0.015, 0.750)	0.081 (0.039, 0.144)	0.341 (0.025, 1.586)	0.027 (0.002, 0.137)
SA 2	*d*_2 → 3_ = 0	25.3	95.3	0.279 (0.017, 0.757)	0.079 (0.038, 0.139)	1.000 (fixed)	0.079 (0.038, 0.139)
	**Sensitivity analyses: Assumptions about between‐trials variation on the 1 → 2 relative treatment effect**						
SA 3	*σ*_1 → 2_ = 0 (*FE*)	27.5	97.2	**‐‐**	0.087 (0.049, 0.142)	0.333 (0.026, 1.551)	0.029 (0.002, 0.140)
SA 4	*σ*_1 → 2_~*Half* − *Normal* (0,0.19^2^)	26.5	96.7	0.152 (0.009, 0.463)	0.085 (0.045, 0.141)	0.324 (0.023, 1.550)	0.027 (0.002, 0.137)
SA 5	*σ*_1 → 2_~*Half* − *Normal* (0,0.50^2^)	24.5	96.0	0.434 (0.029, 1.128)	0.077 (0.031, 0.147)	0.331 (0.025, 1.551)	0.025 (0.002, 0.129)
	**Sensitivity analysis: Assumptions about treatment effects**						
SA 6	$\begin{array}{l} d_{1\to 2}\sim \text{Student}\left(\mathrm{df}=2\right)\\ d_{2\to 3}\sim \text{Student}\left(\mathrm{df}=2\right) \end{array}$	25.5	96.3	0.277 (0.014, 0.764)	0.081 (0.039, 0.143)	0.341 (0.025, 1.571)	0.027 (0.002, 0.137)

† Compare to 24 datapoints

### 4.2 Sensitivity analyses

Models SA 1 and SA 2 appear to fit the data as well as the base‐case model (Table 2). The RRs estimated for SA 1 were consistent with those of the base‐case model (Table 2). As the RR for the 2 → 3 transition for SA 2 is set to 0, RR_1 → 3_ = RR_1 → 2_ (Table 2) but the estimated RR_1 → 2_ is still consistent with that estimated in the base‐case model. No meaningful changes are observed in the estimated between‐study heterogeneity in models SA 1 or 2 compared to the base‐case model.

The treatment effect of IAP also remained consistent when the prior distributions for the heterogeneity of the 1 → 2 transition were varied although the estimates of heterogeneity changed (Table 2). The effect of the different prior distributions on the posterior distributions is shown in the Supplementary Material, S1.

### 4.3 Standard Meta‐analysis models

Table 3 shows the results for the standard meta‐analysis models. The differences between the FE and RE models are negligible for transitions 2 → 3 and 1 → 3. RR_1 → 3_ for the standard meta‐analysis models is consistent with the RR estimated for all multi‐state models except SA 2. The RR estimated for the 1 → 2 transition using standard meta‐analysis is lower than the RR obtained from the multi‐state models. The RR for the 2 → 3 transition is zero. This is due to no cases of EOGBS being observed in the IAP arm in any of the included trials.

**Table 3:** Relative risks and model fit statistics for standard fixed and random effects meta‐analysis models. Between‐study SD for the random effects models are also included. Some studies were included in more than one meta‐analysis

	Number of studies	Number of Datapoints	Fixed effects model		Random effects model		
			Estimate	Residual deviance	Estimate	Between‐study SD	Residual deviance
RR_1 → 2_	7	14	0.059 (0.032, 0.098)	18.3	0.055 (0.029, 0.099)	0.216 (0.010, 0.719)	14.8
RR_2 → 3_^†^	4	8	0.000 (0.000, 4.898)	‐‐	‐‐‐	‐‐‐	‐‐‐
RR_1 → 3_	6	12	0.030 (0.001, 0.171)	10.7	0.029 (0.001, 0.169)	0.216 (0.010, 0.718)	10.7

† The data for the 2 → 3 transition did not allow the use of Bayesian models or the M‐H method to estimate RRs. The RR for the 2 → 3 transition was generated using the exact method^46^ in the exactmeta^47^ package in R 3.4.1.

### 4.4 Checking conflict

There was no evidence of conflict between the overall treatment effects on EOGBS (Direct: 0.01 (4 × 10^−5^, 0.08), Indirect: 0.10 (0.004, 0.70), p‐value: 0.15). The node‐splitting model also appears to fit the data adequately. The residual deviance for 24 datapoints is 23.3 and the DIC is 94.7. The between‐study SD for transition 1 → 2 is 0.29 (0.01, 0.78), similar to *σ*_1 → 2_ obtained in the base‐case model (Table 2). The RRs observed for the node‐splitting models yielded conclusions for the overall treatment effect consistent with those from the multi‐state and standard meta‐analysis models.

## 5 DISCUSSION

We have proposed a model which delivers a single coherent analysis of three nested outcomes and checked the core assumptions to the extent possible. Joint modelling of all the outcomes ensures that all relevant trials provide information on all relative effects of interest, directly or indirectly. The results confirm that the effect of IAP on EOGBS is very strong, eliminating approximately 97.4% (95% CrI: 87.0% ‐ 99.8%) of EOGBS, and suggesting that most of this effect occurs by preventing newborn colonisation. Previous authors^9^, ^31^ also suggest that IAP reduces GBS colonisation in mothers, in turn reducing the transmission to newborns through reduced exposure to GBS during labour.

We have found IAP to be more effective than most previous researchers, but this appears to be mainly because biased estimation methods have been used (Table 4). Smaill (2000)^30^ uses the Peto ‘one‐step’ method^51^ which is biased for unbalanced data or large treatment effects^52^. Benitz (1999)^31^ and Ohlsson (2014)^9^ used Mantel–Haenszel (M‐H) but added a continuity correction factor of 0.5 to cells with zero counts which is not only unnecessary but also incorrect.^53^ The use of continuity correction with sparse data results in bias and poor coverage.^54^, ^55^ The size of these biases, in this case, can be seen by comparing published estimates to the estimates based on Bayesian Fixed Effect and M‐H method without continuity correction (Table 4). Our findings with multi‐state models concur with previous work when appropriate methods were used.

**Table 4:** Results obtained for previous studies

Univariate estimates						
	Transition	Studies included	Method used in study	Pooled estimate reported in study	Mantel–Haenszel (M‐H) OR (no continuity correction)	Bayesian fixed effect OR
Smaill (2000)^30^	1 → 2	Boyer (1986)^28^	Peto OR	0.10 (0.07, 0.14)	0.037 (0.018, 0.074)	0.037 (0.017, 0.069)
		Easmon (1983)^24^				
		Matorras (1991)^23^				
		Morales (1986)^26^				
Smaill (2000)^30^	1 → 3	Boyer (1986)^28^	Peto OR	0.17 (0.07, 0.39)	0.051 (0.007, 0.375)	0.034 (0.001, 0.199)
		Matorras (1991)^23^				
		Morales (1986)^26^				
		Tuppurainen (1989)^27^				
Benitz (1999)^31^	1 → 3	*Allardice (1982)^48^	M‐H OR *with* continuity correction	0.19 (0.07, 0.53)	0.103 (0.023, 0.470)	0.092 (0.012, 0.346)
		Morales (1986)^26^				
		Tuppurainen (1989)^27^				
		Matorras (1991)^23^				
		*Pylipow (1994)^49^				
Allen (1993)^32^	1 → 3	Boyer (1986)^28^	M‐H OR *without* continuity correction	0.03 (0.0013, 0.17)	0.025 (0.004, 0.187)	0.017 (0.001, 0.095)
		*Boyer (1986)^28^				
		*Allardice (1982)^48^				
		*Morales (1987)^50^				
		Morales (1986)^26^				
		Matorras (1991)^23^				
		Tuppurainen (1989)^27^				
Ohlsson (2014)^9^	1 → 3	Boyer (1986) 28	M‐H RR *with* continuity correction	0.17 (0.04, 0.74)	0.097 (0.014, 0.696)^†^	0.062 (0.002, 0.368)^‡^
		Matorras (1991)^23^				
		Tuppurainen (1989)^27^				

* Non‐randomised studies.

† M‐H RR

‡ Bayesian FE RR^35^

NOTE: Reviews did not necessarily extract the same data from each study

The traditional systematic review and meta‐analysis approach forces investigators to choose a “primary” outcome and perform separate analyses for each outcome of interest. This has led to all previous analyses treating the outcomes as unrelated, failing to use all the available evidence or taking into account dependencies and overlaps in the data. One approach would be to treat the 1 → 2 and 1 → 3 outcomes as correlated in a MVNRE meta‐analysis.^5^ This would take account of the within‐ and between‐study correlations, but the analysis would be identical if the outcomes were two different ways of measuring depression, or if one outcome was the risk of stroke, and the other the risk of bleeding. The MPES models presented here, by contrast, are intended to capture structural and clinical relationships, and provide a range of outputs in the way of transition‐specific risk ratios, and model checking, that have a natural clinical interpretation.

The multi‐state models proposed in this paper are related to a much wider set of models for aggregate event history data using rate models and hazard ratios.^56^ These methods include multiple end‐state (or competing risk) models,^57^ models using the Kolmogorov forward equations for data on fully or partially observed Markov processes^58^, ^59^ and models synthesising data on multiple‐outcomes^8^ and over multiple follow‐up times.^7^ In the case of the EOGBS data, although the lack of a time‐element does not allow for event history analysis, the conditional, sequence of outcomes can be used to conduct a coherent analysis.

The most recent Cochrane review,^9^ which concludes “there is a lack of evidence … to recommend IAP” takes all‐cause mortality as the primary outcome, rather than EOGBS, and entirely excludes most of the evidence, which is on newborn colonisation. Only one trial^28^ reports neonatal mortality, with 0/79 deaths in the IAP arm and 2/85 in controls (one death was due to GBS, the other to other causes).

One naturally hesitates before making a treatment recommendation based on such sparse data. However, to treat neonatal EOGBS mortality as if it is unrelated to EOGBS, or to GBS colonisation of the newborn, is reductionism^60^ taken to an illogical extreme. Deaths due to EOGBS can only occur in infants with EOGBS, so prevention of EOGBS is a necessary and sufficient condition to prevent EOGBS‐related mortality. Similarly, to the extent that EOGBS can only occur following newborn colonisation, it would also be reasonable to take newborn colonisation by GBS as a reasonable proxy outcome.

Indeed, if we accept that the effect of IAP on EOGBS mortality is the target parameter, our multi‐state approach readily provides an estimate. If we conservatively assume that there is no effect of treatment on EOGBS mortality in newborns with EOGBS, then the RR of IAP for neonatal mortality due to EOGBS is 0.03 (0.002, 0.13), the same as the effect on EOGBS. As there is evidence that IAP also prevents other early onset disease^61^, ^62^ the effect on all‐cause mortality can only be greater than this.

We have shown how a MPES framework can be used to jointly synthesise all relevant evidence and to check that the underlying assumptions are statistically supported by the evidence available. The starting point for this type of model is an assumption on the clinical relationship between outcomes, which must be clinically plausible and validated by experts.

Similar methods can be applied to trials on fertility treatments where embryo fertilisation, implantation, clinical pregnancy, ongoing pregnancy and birth must follow in order, and the survival of the embryo/fetus at each stage is conditional on survival at the previous stage. Many other multiple outcome evidence structures exist, where a joint synthesis respecting clinical and logical structure provides a more robust basis for systematic review and decision making,^6^, ^7^, ^8^, ^58^ especially if formal methods such as cost‐effectiveness analysis are employed.^63^, ^64^

## FUNDING

SA and SD were supported by the Medical Research Council, UK (grant number MR/M005232/1).

## CONFLICT OF INTEREST

None.

## Supporting information

Figure S1. Prior and Posterior distribution for the between‐study standard deviation for the 1➔2 transition. The prior distributions used were: (a) Half‐norman (0,0.32^2^), (b) Half‐Norman (0,0.19^2^), (c) Half Normal (0,0.50^2^)

Figure S2. Prior and posterior distributions for the treatment effect for the 1➔2 transition, d [1]. The prior distributions were (a) Normal (0,1000) for the base‐case model and (b) the corresponding Student‐t distribution with 2 degrees of freedom for the SA 6 model.

Figure S3. Prior and posterior distributions for the treatment effect for the 2➔3 transition, d [2]. The prior distributions were (a) Normal (0,10) for the base‐case model and (b) the corresponding Student‐t distribution with 2 degrees of freedom for the SA model.

Data S4: Supplementary Information

## Data Availability

Data sharing is not applicable to this article as no new data were created or analysed in this study.
