# Supplementary figures and images for: Joint synthesis of conditionally related multiple outcomes makes better use of data than separate meta‐analyses
Source: Res Synth Methods. 2019 Nov 10;11(4):496–506. doi: 10.1002/jrsm.1380 (PMC7383979; doi:10.1002/jrsm.1380)

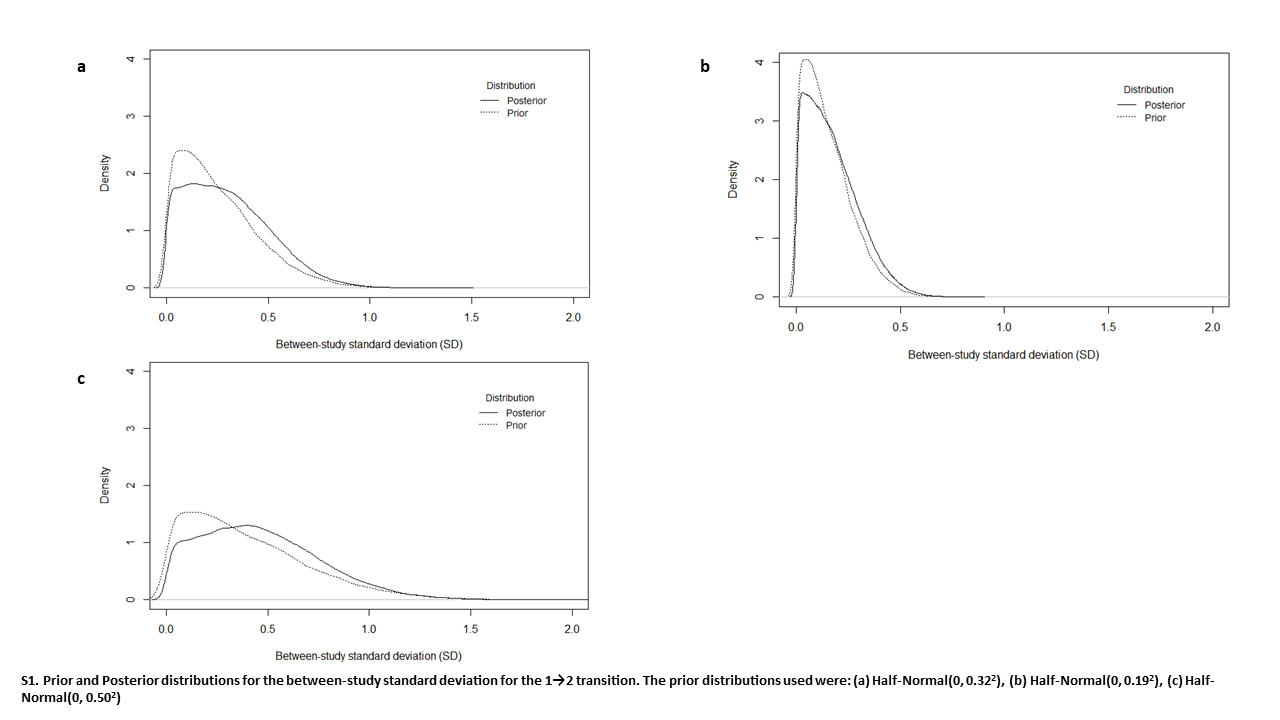

Supplement: Supplementary file 1 — Figure S1. Prior and Posterior distribution for the between‐study standard deviation for the 1➔2 transition. The prior distributions used were: (a) Half‐norman (0,0.322), (b) Half‐Norman (0,0.192), (c) Half Normal (0,0.502) [file JRSM-11-496-s001.tif]

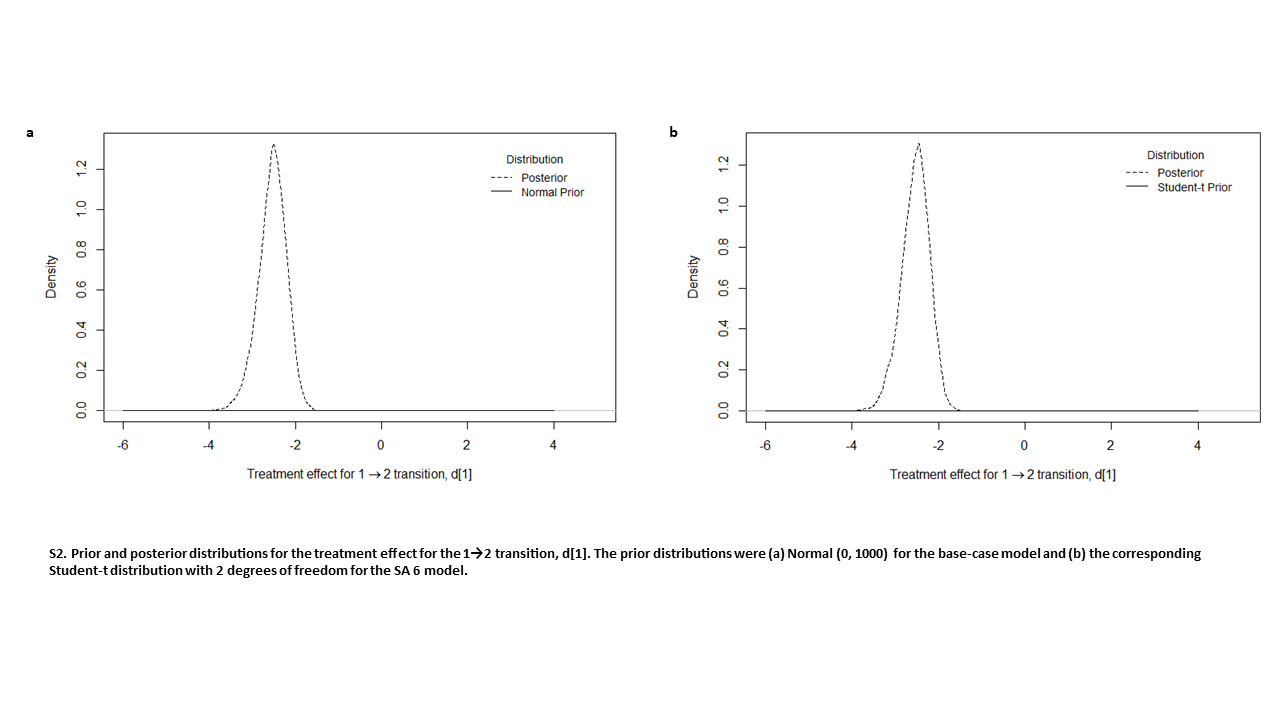

Supplement: Supplementary file 2 — Figure S2. Prior and posterior distributions for the treatment effect for the 1➔2 transition, d [1]. The prior distributions were (a) Normal (0,1000) for the base‐case model and (b) the corresponding Student‐t distribution with 2 degrees of freedom for the SA 6 model. [file JRSM-11-496-s002.tif]

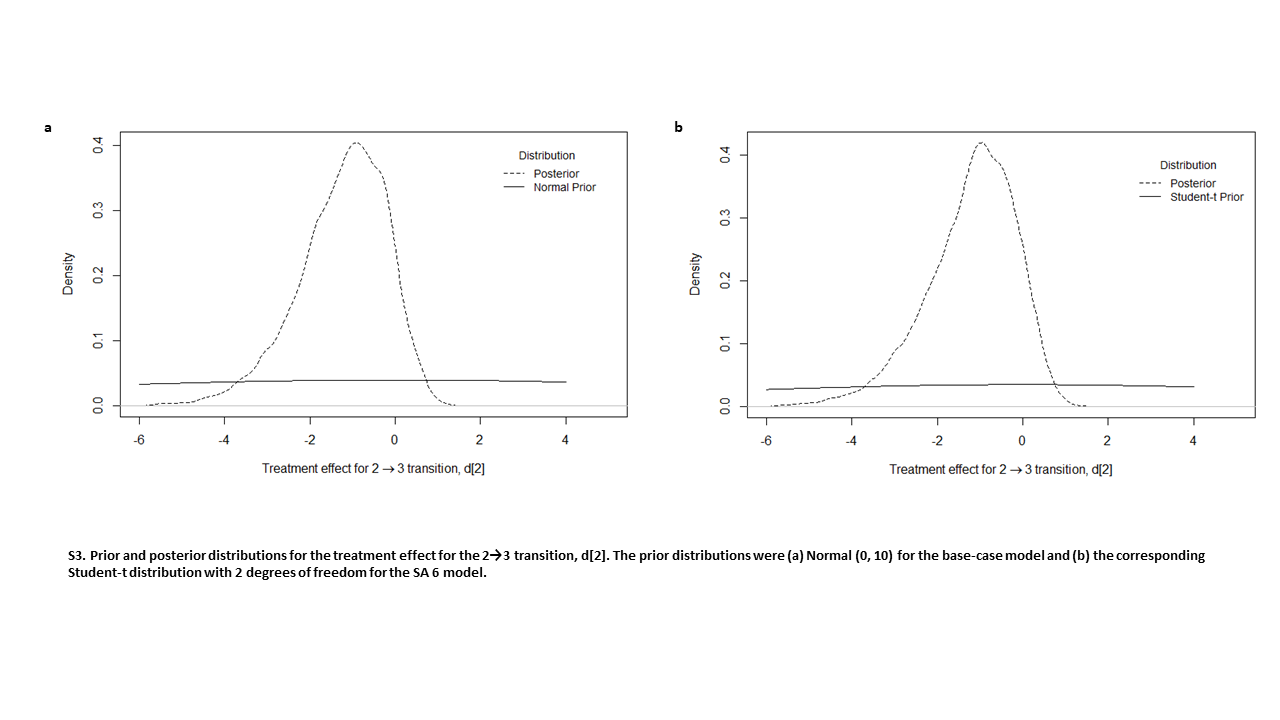

Supplement: Supplementary file 3 — Figure S3. Prior and posterior distributions for the treatment effect for the 2➔3 transition, d [2]. The prior distributions were (a) Normal (0,10) for the base‐case model and (b) the corresponding Student‐t distribution with 2 degrees of freedom for the SA model. [file JRSM-11-496-s003.tif]
