# Supplementary material for: Joint synthesis of conditionally related multiple outcomes makes better use of data than separate meta‐analyses
Source: Res Synth Methods. 2019 Nov 10;11(4):496–506. doi: 10.1002/jrsm.1380 (PMC7383979; doi:10.1002/jrsm.1380)
Supplement: Supplementary file 4 — Data S4: Supplementary Information [file JRSM-11-496-s004.docx]

**Supplementary File 4: WinBUGS Code**

**Data**

The same data was used for the base-case model, node-splitting model and all the sensitivity analyses

r[,1] n[,1] r[,2] n[,2]

**# 1 => 2**

46 82 2 69 # Boyer (1982)

13 37 1 43 # Boyer (1983)

40 79 8 85 # Boyer (1986)

24 56 2 54 # Matorras (1991)

17 49 0 38 # Easmon (1983)

14 24 0 34 # Yow (1979)

**# 2 => 3**

4 46 0 2 # Boyer (1982)

1 13 0 1 # Boyer (1983)

5 40 0 8 # Boyer (1986)

3 24 0 2 # Matorras (1991)

**# 1=>3**

4 111 1 88 # Tuppurainen (1989)

2 128 0 135 # Morales (1986)

END

**Initial values**

The same initial values were used for all multi-state models. The initial values for all the other parameters were generated by WinBUGS

# chain 1

list(d=c(0,0,NA))

# chain 2

list( d=c(-1,-3,NA))

# chain 3

list( d=c(2,6,NA))

# chain 4

list( d=c(1,-3,NA))

**1. Base-case Model**

model{

for (i in 1:12){ # loop through all trials

mu[i] <- log(p[i,1])

p[i,1] ~ dunif(0,1) # vague priors for all trial baselines

for (k in 1:2){

r[i,k] ~ dbin(p[i,k],n[i,k]) # binomial likelihood

rhat[i,k] <- p[i,k] * n[i,k] # expected value of the numerators

dev[i,k] <- (2*(r[i,k]*(log(r[i,k])-log(rhat[i,k])) + (n[i,k]-r[i,k])*(log(n[i,k]-r[i,k])

- log(n[i,k]-rhat[i,k])))) # deviance contribution

}

}

# 1=> 2 model

for (i in 1:6){

log(p[i,2]) <- mu[i] + min(delta[i],-mu[i])

delta[i] ~ dnorm(d[1],tau) # 1=>2 trial-specific effects

}

d[1] ~ dnorm(0,.001) # vague priors for mean 1=>2 effect

sd ~ dnorm(0,prec) I(0,) # Half-normal(0, 0.32^2) prior for SD

prec <- pow(0.32,-2)

tau <- pow(sd, -2)

# 2=> 3 model

for (i in 7:10){ log(p[i,2]) <- mu[i] + min(d[2],-mu[i]) }

d[2] ~ dnorm(0,.1) # weakly informative prior for 2=>3 effect

d[3] <- d[1] + d[2] # Indirect 1=>3 effect

# 1=> 3 model

for (i in 11:12){

log(p[i,2]) <- mu[i] + min(delta2[i],-mu[i])

delta2[i] ~ dnorm(d[3], tau)

}

totresdev <- sum(dev[,]) # total residual deviance

for (m in 1:3) { log(rr[m]) <- d[m] } # calculate RR

}

**2. Node-splitting Model**

model{

for (i in 1:12){ # loop through all trials

mu[i] <- log(p[i,1])

p[i,1] ~ dunif(0,1) # vague priors for all trial baselines

for (k in 1:2){

r[i,k] ~ dbin(p[i,k],n[i,k]) # binomial likelihood

rhat[i,k] <- p[i,k] * n[i,k] # expected value of the numerators

dev[i,k] <- (2*(r[i,k]*(log(r[i,k])-log(rhat[i,k])) + (n[i,k]-r[i,k])*(log(n[i,k]-r[i,k])

- log(n[i,k]-rhat[i,k])))) # deviance contribution

}

}

# 1=> 2 model

for (i in 1:6){

log(p[i,2]) <- mu[i] + min(delta[i],-mu[i])

delta[i] ~ dnorm(d[1],tau) # 1=>2 trial-specific effects

}

d[1] ~ dnorm(0,.001) # vague priors for mean 1=>2 effect

sd ~ dnorm(0,prec) I(0,) # Half-normal(0, 0.32^2) prior for SD

prec <- pow(0.32,-2)

tau <- pow(sd, -2)

# 2=> 3 model

for (i in 7:10){

log(p[i,2]) <- mu[i] + min(d[2],-mu[i])

}

d[2] ~ dnorm(0,.1) # weakly informative prior for 2=>3

# 1=> 3 model

for (i in 11:12){

log(p[i,2]) <- mu[i] + min(delta2[i],-mu[i])

delta2[i] ~ dnorm(d[3], tau)

}

d[3] ~ dnorm(0, 0.0001) # Direct 1 => 3

d[4] <- d[1] + d[2] # Indirect 1 => 3

# Checking for conflict

p.xval <- step(d[3]-d[4]) # Direct 1=>3 - Indirect 1=>3

totresdev <- sum(dev[,]) # total residual deviance

for (m in 1:4) { log(rr[m]) <- d[m] } # calculate RR

}

**3. Sensitivity Analyses**

The changes that were made to the base-case model code for each sensitivity analysis are given below.

**3.1. Modelling Assumptions**

**Sensitivity Analysis 1: Fixed Effect Model for the 1 => 3 Transition**

Replace the following section of the code:

*# 1=> 3 model*

*for (i in 11:12){ log(p[i,2]) <- mu[i] + min(delta2[i],-mu[i])*

*delta2[i] ~ dnorm(d[3], tau) }*

with:

*# 1=> 3 model*

*for (i in 11:12){ log(p[i,2]) <- mu[i] + min(d[3],-mu[i]) }*

*d[3] <- d[1] + d[2] # synthesis of 1=>3 effect*

**Sensitivity Analysis 2: Transition 2 => 3 Treatment Effect = 0**

Replace the following section of the code:

*# 2=> 3 model*

*for (i in 7:10){ log(p[i,2]) <- mu[i] + min(d[2],-mu[i]) }*

*d[2] ~ dnorm(0,.1) # weakly informative prior for 2=>3 effect*

*d[3] <- d[1] + d[2] # Indirect 1=>3 effect*

*# 1=> 3 model*

*for (i in 11:12){ log(p[i,2]) <- mu[i] + min(delta2[i],-mu[i])*

*delta2[i] ~ dnorm(d[3], tau) }*

with:

*# 2=> 3 model*

*for (i in 7:10){ log(p[i,2]) <- mu[i] }*

*d[2] <-0*

*# 1=> 3 model*

*for (i in 11:12){ log(p[i,2]) <- mu[i] + min(d[3],-mu[i]) }*

*d[3] <- d[1] + d[2] # synthesis of 1=>3 effect*

**3.2 Assumptions about between-trial variation on the 1 => 2 transition**

**Sensitivity Analysis 3: Fixed Effects for the 1 => 2 Transition**

Replace the following section of the code:

*# 1=> 2 model*

*for (i in 1:6){log(p[i,2]) <- mu[i] + min(delta[i],-mu[i])*

*delta[i] ~ dnorm(d[1],tau) # 1=>2 trial-specific effects }*

*d[1] ~ dnorm(0,.001) # vague priors for mean 1=>2 effect*

with:

*# 1=> 2 model*

*for (i in 1:6){log(p[i,2]) <- mu[i] + min(d[1],-mu[i]) }*

*d[1] ~ dnorm(0,.001) # vague priors for mean 1=>2 effect*

**Sensitivity Analysis 4: Half-normal (0, 0.19^2^) Prior**

Replace the following section of the code:

*prec <- pow(0.32,-2)*

with:

*prec <- pow(0.19,-2)*

**Sensitivity Analysis 5: Half-normal (0, 0.50^2^) Prior**

Replace the following section of the code:

*prec <- pow(0.32,-2)*

with:

*prec <- pow(0.19,-2)*

**3.3. Assumptions about treatment effects**

**Sensitivity Analysis 6: Student-t Prior for 1=>2 and 2=>3 Transitions**

Replace the following sections of the code, changing the prior distributions:

*d[1] ~ dnorm(0,.001) # vague priors for mean 1=>2 effect*

with:

*d[1] ~ dt(0,.001,2) # vague priors for mean 1=>2 effect*

And replace:

*d[2] ~ dnorm(0,.1) # weakly informative prior for 2=>3 effect*

with:

*d[2] ~ dt(0,.1,2) # weakly informative prior for 2=>3 effect*

**4. Pair-wise Meta-Analysis Models**

The same code was used for the three transitions and the same initial values were used. The initial values that are not given were generated in WinBUGS. The data differed for each transition.

**Initial Values:**

list(d=0)

list(d=3)

list(d=-1)

list(d=5)

**4.1. Fixed Effect Model**

model{

for (i in 1:nt){

mu[i] <- log(pA[i])

pA[i] ~ dunif(0,1)

rA[i] ~ dbin(pA[i], nA[i])

rB[i] ~ dbin(pB[i], nB[i])

rhatA[i] <- pA[i] * nA[i]

rhatB[i] <- pB[i] * nB[i]

log(pB[i]) <- mu[i] + min(d,-mu[i])

devA[i] <- (1-equals(i,nt))*(2*(rA[i]*(log(rA[i])-log(rhatA[i])) + (nA[i]-rA[i])*(log(nA[i]-rA[i])

- log(nA[i]-rhatA[i]))))

devB[i] <- (1-equals(i,nt))*(2*(rB[i]*(log(rB[i])-log(rhatB[i])) + (nB[i]-rB[i])*(log(nB[i]-rB[i])

- log(nB[i]-rhatB[i]))))

resdev[i] <- devA[i] +devB[i]

}

totresdev <- sum(resdev[]) #Total Residual Deviance

d ~ dnorm(0,.0001)

RR<-exp(d)

}

**4.2. Random Effects Model**

model{

for (i in 1:nt){

mu[i] <- log(pA[i])

pA[i] ~ dunif(0,1)

rA[i] ~ dbin(pA[i], nA[i])

rB[i] ~ dbin(pB[i], nB[i])

rhatA[i] <- pA[i] * nA[i]

rhatB[i] <- pB[i] * nB[i]

log(pB[i]) <- mu[i] + min(delta[i],-mu[i])

delta[i] ~ dnorm(d, prec)

devA[i] <- (1-equals(i,nt))*(2*(rA[i]*(log(rA[i])-log(rhatA[i])) + (nA[i]-rA[i])*(log(nA[i]-rA[i])

- log(nA[i]-rhatA[i]))))

devB[i] <- (1-equals(i,nt))*(2*(rB[i]*(log(rB[i])-log(rhatB[i])) + (nB[i]-rB[i])*(log(nB[i]-rB[i])

- log(nB[i]-rhatB[i]))))

resdev[i] <- devA[i] +devB[i]

}

totresdev <- sum(resdev[]) #Total Residual Deviance

d ~ dnorm(0,.0001)

RR<-exp(d)

tau~dunif(0,1)

tau.sq <- tau*tau

prec <- 1/(tau.sq)

}

***# For the 1=>2 Transition***

**Data:**

list(nt=7)

rA[] nA[] rB[] nB[]

46 82 2 69 # Boyer (1982)

13 37 1 43 # Boyer (1983)

40 79 8 85 # Boyer (1986)

24 56 2 54 # Matorras (1991)

17 49 0 38 # Easmon (1983)

14 24 0 34 # Yow (1979)

59 128 0 135 # Morales (1986)

END

***# For the 2=>3 Transition***

**Data**

list(nt=4)

rA[] nA[] rB[] nB[]

4 46 0 2 # Boyer (1982)

1 13 0 1 # Boyer (1983)

5 40 0 8 # Boyer (1986)

3 24 0 2 # Matorras (1991)

END

***# For the 1=>3 Transition***

**Data**

list(nt=6)

rA[] nA[] rB[] nB[]

4 82 0 69 # Boyer (1982)

1 37 0 43 # Boyer (1983)

3 56 0 54 # Matorras (1991)

4 111 1 88 # Tuppurainen (1989)

2 128 0 135 # Morales (1986)

5 79 0 85 # Boyer (1986)

END
